# Seminal plasma removal for medium-term preservation of ram sperm at 5 °C

**DOI:** 10.1186/s12917-024-04214-5

**Published:** 2024-08-12

**Authors:** Marta Neila-Montero, Mercedes Alvarez, Marta F. Riesco, Cristina Soriano-Úbeda, Rafael Montes-Garrido, Cristina Palacin-Martinez, Paulino de Paz, Luis Anel, Luis Anel-Lopez

**Affiliations:** 1 Itra-ULE, INDEGSAL, University of León, Campus de Vegazana S/N, León, 24071 Spain; 2 Animal Reproduction and Obstetrics, Department of Veterinary Medicine, Surgery and Anatomy, University of León, Campus de Vegazana S/N, León, 24071 Spain; 3 Cellular Biology, Department of Molecular Biology, University of León, Campus de Vegazana S/N, León, 24071 Spain; 4 Anatomy, Department of Veterinary Medicine, Surgery and Anatomy, University of León, Campus de Vegazana S/N, León, 24071 Spain

**Keywords:** Chilled semen, Cooled semen, Epididymal sperm, Ovine, Semen preservation

## Abstract

This study aimed to investigate if washing ram sperm from seminal plasma (SP) could be an effective tool to extend sperm lifespan in medium-term preservation in liquid form to optimize ovine artificial insemination protocols. To this end, in Experiment 1 SP was added to a sperm model without previous contact with this substance (ram epididymal sperm) at the beginning or the end of a 48-hour preservation protocol at 5 °C (*n* = 13). Sperm motility and kinetic parameters and sperm functionality in terms of sperm viability, apoptosis, mitochondrial activity and reacted acrosomes were assessed after 6 h of storage at 15 °C (standard liquid preservation method) and 24 and 48 h at 5 °C. Extended sperm showed better results after 48 h when stored in the absence than in the presence of SP in most sperm quality parameters. Moreover, the final SP supplementation of this experimental group resulted in the highest sperm motility and kinetic parameters, viability and mitochondrial activity. These results suggested that initial SP deprivation could be beneficial in a medium-term ram sperm preservation protocol in liquid form, as well as a final supplementation. Therefore, we conducted Experiment 2 to evaluate the effect of SP removal from freshly ejaculated ram semen under the same storage conditions as in Experiment 1 (*n* = 12). Surprisingly, SP withdrawal impaired sperm functionality, leading to increased apoptosis and decreased mitochondrial activity after 24 and 48 h at 5 °C. Conversely, SP supplementation at the end of the preservation protocol of the ejaculate processed as usual had a positive effect on sperm quality and fertility. To summarize, SP absence was beneficial for a medium-term preservation protocol (up to 48 h at 5 °C) of ram epididymal sperm, but the same preservation protocol for ram ejaculated sperm revealed a possible failure of the SP removal method in avoiding the sperm-SP interaction effect. Meanwhile, SP supplementation of ram semen at the end of the preservation protocol increased in vitro sperm quality and fertility after artificial insemination.

## Introduction

Artificial insemination is an essential tool in animal breeding programs. However, it is not widespread in sheep due to the variability of its fertility results and the specific problems presented by its application [1–4]. From a methodological point of view, the obstacle lies in the high structural complexity of the ewe cervix, which prevents deep artificial insemination and reduces the efficiency of the technique [5, 6]. Such a long fibrous tubular structure with inner rings requires the use of laparoscopic intrauterine artificial insemination to ensure adequate fertility when frozen-thawed semen is used [7], but this route of sperm application has some limitations, including high cost and time-consuming [8]. For that reason, cervical artificial insemination with cooled semen (15 °C) is the most commonly used method in commercial programs due to its simplicity and satisfactory results [9, 10]. Nevertheless, this artificial insemination procedure also presents several problems, such as hygienic risks and limited semen transport time due to a short fertile lifespan of ram sperm (6–8 h from the collection), leading to dependence on a nearby reproduction center for the preparation of semen doses on the day of artificial insemination [11]. Therefore, new strategies should be designed to optimize artificial insemination procedures in this species to increase their implementation. In this sense, the development of a medium-term sperm preservation method in liquid form (48 h at 5 °C) would be useful, allowing better management of the reproduction centers and facilitating the use of the technique by the farmer. Especially relevant here is the role of the seminal plasma (SP), a mixture of secretions from testes, epididymides, and accessory glands that merges with sperm from the tail of the epididymis at ejaculation, resulting in semen [12]. Because in natural mating sperm are quickly separated from the SP in the female reproductive tract, it was initially thought to serve exclusively as a sperm transport medium [13]. Now, it is recognized as a controversial substance in sperm preservation due to its complex composition, which varies even among closely related species [14]. To date, several studies have documented the protective effect of SP from stress conferred by sperm processing and preservation by cooling or freezing in ram [15–21], but also in other species such as bull [22, 23], red deer [24], boar [25, 26], stallion [27], and human [28–30]. Conversely, detrimental effects of SP on sperm motility and survival after freezing-thawing have also been reported in equine [31], porcine [32], and certainly ovine sperm [33, 34]. SP contains proteins and low molecular weight compounds such as Na^+^, K^+^, Ca^2+^, and heavy metals that could be counterproductive and reduce sperm survival in ejaculate preservation [35–37]. In addition, the influence of SP in sperm capacitation and ageing has been widely discussed by different authors confronting contrary positions [38–41]. Thus, it has been recently suggested that SP removal may be beneficial for the liquid storage of sperm in most farm animals [42]. However, reports regarding SP withdrawal for cooling sperm are scarce. The positive effect of SP removal on cooled preservation has already been verified in sperm of stallions in terms of membrane stability [43] and in boars through enhanced acrosome integrity and *in vivo* fertility rate [44]. In contrast, removal of SP did not seem advantageous on cooled donkey sperm [45]. For ram sperm, there are only three studies assessing the effect of the removal of SP, with conflicting results [46–48].

Thus, to investigate if washing ram sperm from SP could be an effective tool to extend sperm lifespan in medium-term preservation in liquid form, we assessed: (1) sperm quality of epididymal sperm after SP addition, and (2) sperm quality and fertility after artificial insemination of freshly ejaculated ram semen deprived of SP using an optimized centrifugation protocol previously designed by our research group [49].

## Materials and methods

### Reagents and media

All products used in this study were of reagent grade or higher and were procured from Sigma Aldrich (Saint Louis, MI, USA) unless specified differently.

INRA 96^®^, an extender based on modified Hanks’ salts with 67 mM glucose, 126 mM lactose, native phosphocaseinate (27 g/L), potassium penicillin G (38 mg/mL), gentamicin (105 mg/mL), and amphotericin B (0.315 mg/mL), was acquired from IMV Technologies (L’Aigle, France).

Fluorescence probe Zombie Violet™ Fixable Viability Kit was purchased from BioLegend (San Diego, CA, USA), CellEvent™ Caspase-3/7 Green Detection Reagent and Lectin PNA from *Arachis hypogaea* (peanut) Alexa Fluor™ 488 Conjugate were sourced from ThermoFisher (Waltham, MA, USA), and CellROX™ Deep Red Reagent was obtained from Invitrogen (Eugene, OR, USA). Stock solution of Zombie Violet™ was prepared in Dimethyl Sulfoxide (DMSO) following manufacturer instructions (100 µL DMSO was added to one vial of lyophilized Zombie Violet™ dye), and stock solution of PNA Alexa Fluor™ 488 Conjugate was prepared in double-distilled water at a concentration of 1 mg/1 mL (1 mM).

### Animals

A total of 33 Assaf rams aged between two and eight years, of proven fertility and trained for semen collection by artificial vagina regularly (two collections two days per week) were the subject of the experiments after authorization from the Sheep and Goat Selection and Genetic Improvement Center of Castilla y León (Ovigén, Villalazán, Zamora, Spain). Males were housed and fed a standard balanced diet at the Animal Selection and Reproduction Center of Junta de Castilla y León (CENSYRA, Villaquilambre, León, Spain) and Ovigén. Animals for the collection of epididymal sperm were selected from rams that were to be slaughtered for meat production due to genetic quality reasons in a local slaughterhouse certified for this purpose and in compliance with European and Spanish Regulations (1099/2009/EU and RD/37/2014, respectively). Moreover, 174 adult ewes inscribed in the Selection and Genetic Improvement Program of the Assaf breed and supervised by the National Association of Assaf Breeders (ASSAF.E) were included in the artificial insemination protocol before the consent of the farmers.

### Seminal plasma collection

Two ejaculates per male were collected by an artificial vagina of an inner sleeve temperature of 40 °C (IMV Technologies, L’Aigle, France) in the presence of a female decoy during the breeding season. Semen samples were maintained in a water bath (30 °C) during the initial evaluation of semen quality. Ejaculate volume was determined using the graduation marks of the collection tube. Mass motility was assessed with a subjective score of 0–5 by a microscope equipped with a warmed stage programmed at 37 °C (Leica DM LB, Meyer Instruments, Houston, TX, USA) using an X4 objective. Sperm concentration was analyzed by a cell counter (NucleoCounter SP-100, ChemoMetec, Allerod, Denmark). After verifying the good quality of the semen samples (volume: ≥ 0.5 mL; mass motility: ≥ 4; sperm concentration: ≥ 3 × 10^9^ sperm/mL), both ejaculates from each male were mixed and centrifuged at 10,000 × *g* for 15 min at 4 °C. SP was collected, checked for purity using phase contrast microscopy, and frozen at −80 °C until use.

### Sperm collection and processing

#### Epididymal sperm

**Fig. 1:**
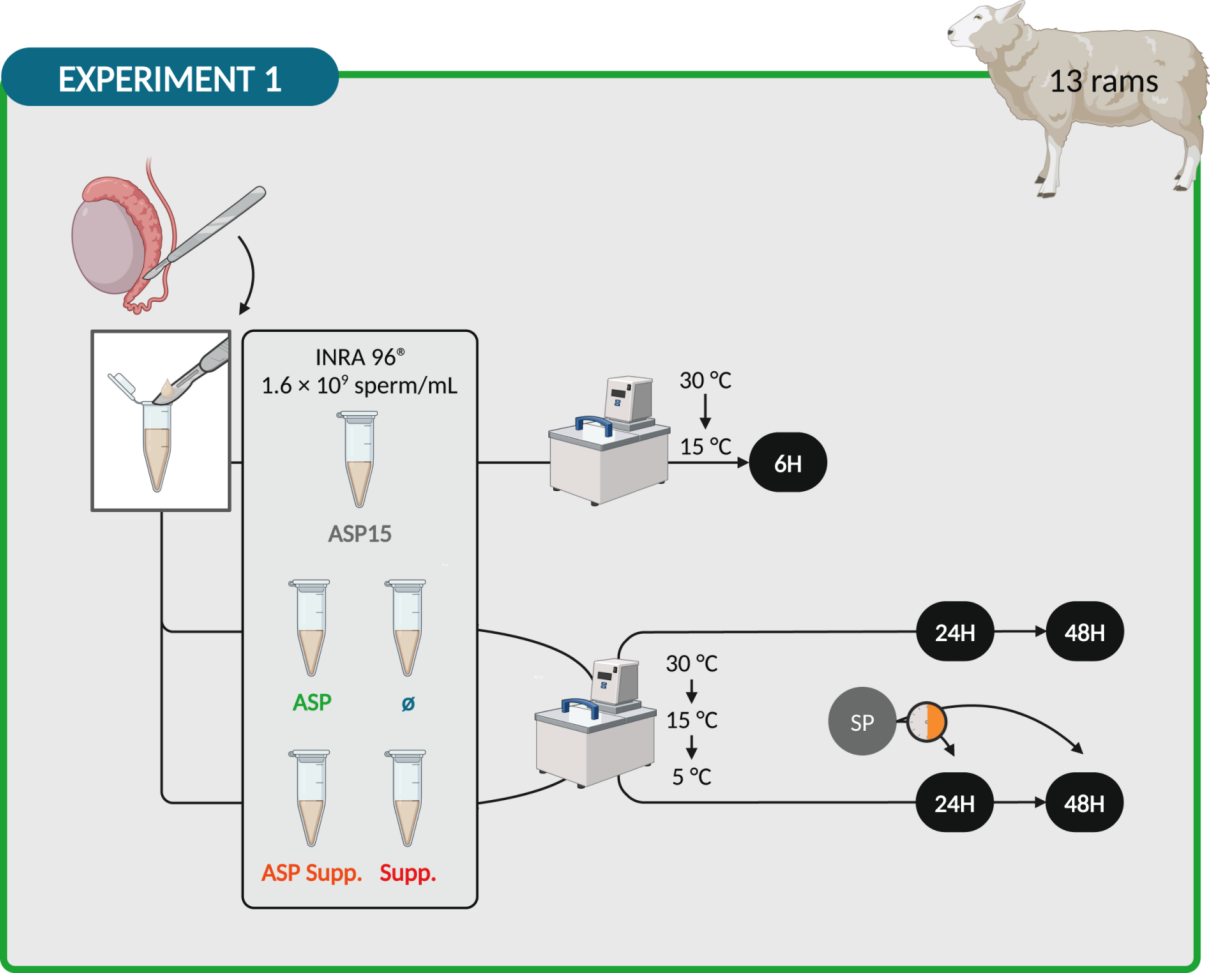
Design of Experiment 1: Seminal plasma addition to epididymal sperm. Created with BioRender.com

Epididymal sperm were obtained from 13 males as previously described by Neila-Montero *et al.* [50]. The week after SP collection, testicles obtained from the local slaughterhouse were transported to our laboratory in a portable refrigerator at 22 °C (CoolFreeze CF-25, Dometic Group, Stockholm, Sweden). On arrival, about 30 min post-mortem, epididymides were dissected and cleaned of connective tissue and superficial blood vessels to avoid blood contamination. Sperm were collected through several incisions on the cauda epididymis with a surgical blade taking the emerging fluid. Epididymal sperm were split into five aliquots to establish the different experimental groups. The first aliquot (ASP15) was prepared by adding 30% (v/v) autologous SP (derived from the same ram as the sperm) and INRA 96^®^ to the epididymal sperm until a final concentration of 1.6 × 10^9^ sperm/mL was achieved. After that, this aliquot was refrigerated in a programmable bath (CC-K8, Huber, Germany) using a rate of –0.5 °C/min from 30 °C to 15 °C and stored at 15 °C for 6 h in an attempt to simulate an ejaculate processed in the usual form [50]. The second aliquot (ASP) was made in the same way but was refrigerated in a programmable bath using a rate of −0.5 °C/mine from 30 °C to 15 °C and −0.25 °C/min from 15 °C to 5 °C and stored at 5 °C for 24 and 48 h (preservation method also used for the third, fourth and fifth aliquots). The third (Ø) was generated by diluting the epididymal sperm to a final concentration of 1.6 × 10^9^ sperm/mL in INRA 96^®^. Finally, fourth (ASP Supp.) and fifth (Supp.) aliquots were produced as second and third, but 30 min before sperm quality assessment they were supplemented with 30% autologous SP (Fig. 1). Autologous SP was chosen because it is more beneficial than homologous SP, whose composition considerably differs among donor males [51].

#### Ejaculated sperm

**Fig. 2:**
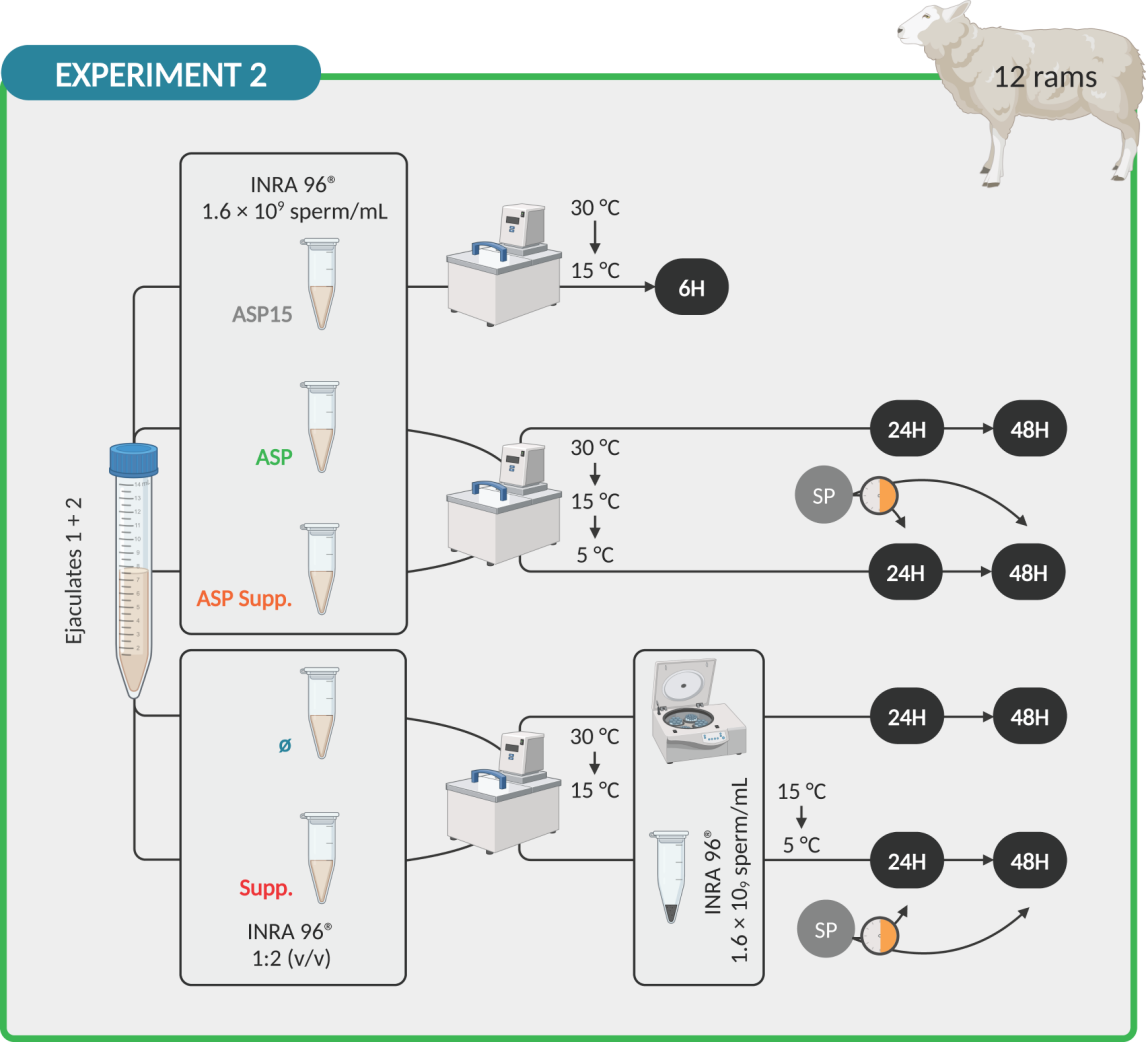
Design of Experiment 2: Seminal plasma removal of ejaculated sperm. Created with BioRender.com

The week following SP collection, ejaculates (two per male) were collected from 12 males using an artificial vagina during the breeding season. Only ejaculates of good quality of each ram were mixed and used. The semen was divided into five aliquots to establish experimental groups equivalent to those of epididymal sperm. For the first aliquot (ASP15), semen was diluted in INRA 96^®^ to a final concentration of 1.6 × 10^9^ sperm/mL, refrigerated in a programmable bath using a rate of −0.5 °C/min from 30 °C to 15 °C and stored at 15 °C for 6 h (standard liquid preservation method). The second (ASP) consisted of the same as the first but refrigerated in a programmable bath using a rate of –0.5 °C/min from 30 °C to 15 °C and −0.25 °C/min from 15 °C to 5 °C and stored at 5 °C for 24 and 48 h as the third, fourth and fifth aliquots. The third (Ø) was created by removing SP by centrifugation of the ejaculate at 1,200 × *g* for 10 min at 15 °C as described by Neila-Montero *et al.* [49] and diluting the resultant sperm pellet in INRA 96^®^ to obtain a final concentration of 1.6 × 10^9^ sperm/mL. Finally, as for epididymal sperm, fourth (ASP Supp.) and fifth (Supp.) aliquots were obtained as second and third but were supplemented with 30% autologous SP 30 min before the sperm quality evaluation (Fig. 2).

#### Artificial insemination protocol

Two ejaculates per male were obtained from 8 Assaf rams by artificial vagina during the breeding season. Only ejaculates with volume ≥ 0.5 mL, mass motility ≥ 4 and sperm concentration ≥ 3 × 10^9^ sperm/mL were processed. Both ejaculates from each ram were mixed and divided into five aliquots to create the same experimental groups as for ejaculated sperm: ASP15, ASP, Ø, ASP Supp., and Supp. Semen was packed into 0.25 mL plastic straws and transported to farms at 15 °C or 5 °C in a portable refrigerator. Cervical artificial insemination was performed 6 and 24 h after collection of the semen according to the experimental group (ASP15 at 6 h, and ASP, Ø, ASP Supp., and Supp. at 24 h).

### Sperm quality evaluation

#### Sperm motility and kinetic parameters by a CASA system

Sperm motility and kinetic parameters were assessed using Computer-Assisted Sperm Analysis (CASA) (Sperm Class Analyzer^®^ –SCA– software V 6.3.0.59, Microptic S.L., Barcelona, Spain) set to capture at 100 frames/s a total of 50 frames and particles with an area of 20–70 µm^2^. Samples of the different experimental groups were diluted to a final concentration of 25 × 10^6^ sperm/mL in a TES-Tris-Fructose medium supplemented with 1% clarified egg yolk (320 mOsm/kg, pH 7.2) and warmed on a 37 °C plate for 5 min. A drop of 5 µL of the diluted samples was placed into a Makler counting cell chamber (10 μm depth; Sefi Medical Instruments, Haifa, Israel). Samples were examined with an X10 negative phase contrast objective in a microscope (Eclipse E400, Nikon, Tokyo, Japan) equipped with a BASLER acA1300-200uc digital camera (Basler Vision Technologies, Ahrensburg, Germany) and a warmed stage at 37 °C. At least 400 sperm from four randomly selected fields were captured and analyzed. Reported kinetics parameters were curvilinear velocity (VCL, µm/s), linearity (LIN, %), and amplitude of the lateral displacement of the sperm head (ALH, µm). Total motility (TM), progressive motility (PM), and fast progressive motility (FPM) were defined as the percentage of sperm with VCL > 15 μm/s, 45 μm/s, and 75 μm/s, respectively [52].

#### Sperm functionality by flow cytometry

Flow cytometry analyses were conducted in a MACSQuant Analyzer 10 (Miltenyi Biotech, Bergisch Gladbach, Germany) equipped with three lasers emitting at 405, 488, and 635 nm (violet, blue and red, respectively) and ten photomultiplier tubes. The system was controlled using MACS Quantify™ software (Miltenyi Biotech, Bergisch Gladbach, Germany), recording 40,000 events per sample and at least 20,000 sperm at a 200–300 cells/s flow rate. Data were analyzed using FlowJo™ V 10.8.1 (Ashland, Wilmington, DE, USA).

##### Sperm viability, apoptosis and mitochondrial activity

The staining protocol previously described by Riesco and her colleagues [53] was used. Briefly, work solutions of fluorescent probes were prepared in phosphate-buffered saline (PBS) (300 mOsm/kg, pH 7.2) at the following concentrations: 1 µL/1 mL for Zombie Violet™ stock solution, 1 µL/10 µL for CellEvent™ Caspase-3/7 Green (0.2 mM), and 1 µL/10 µL for CellROX™ Deep Red (0.25 mM). Samples of the different experimental groups were diluted at 2 × 10^6^ sperm/mL in PBS to wash the cells by a short centrifugation spin (15 s; MiniSpin plus, Eppendorf, Hamburg, Germany) with the removal of the supernatant. Then, cells were incubated at room temperature in the dark for 30 min with 96 µL of Zombie Violet™ Fixable Viability Kit work solution (plasma membrane integrity probe) (1:1000 final dilution), 2 µL of CellEvent™ Caspase-3/7 Green Detection Reagent work solution (apoptosis marker) (4 µM final concentration) and 2 µL of CellROX™ Deep Red Reagent work solution (reactive oxygen species –ROS– content labelling) (5 µM final concentration). After that, another washing step was performed to detain cell staining, and the pellet was resuspended in 1 mL PBS, carrying out immediate flow cytometry analysis. Violet, green, and red fluorescence were detected in V1 (excitation 405 nm, emission 450/50 nm), B1 (excitation 488 nm, emission 525/50 nm), and R1 (excitation 635 nm, emission 655–730 nm (655LP + split 730)), respectively. Viability was measured as the percentage of sperm with intact plasmalemma (sperm low stained with Zombie Violet™), apoptosis using the sperm subgroup with active caspases 3 and 7 (sperm stained with CellEvent™ Caspase-3/7 Green), and mitochondrial activity by the subpopulation of sperm with high ROS content (CellROX™ Deep Red positive sperm).

##### Acrosomal status

The acrosomal status of sperm was evaluated using Lectin PNA from *Arachis hypogaea* (peanut) Alexa Fluor™ 488 Conjugate. The work solution was prepared in PBS at a concentration of 1 µL stock solution/1 mL (1 µM) [54]. Sperm samples (2 × 10^6^ sperm/mL) were washed as in the previous section and incubated at room temperature for 30 min in the dark with 100 µL of PNA Alexa Fluor™ 488 Conjugate work solution (1 µM final concentration). A final wash and resuspension of the stained sample in PBS were performed for analysis by flow cytometry. Data corresponding to the green fluorescence were recorded in B1, and stained sperm with the PNA Alexa Fluor™ 488 Conjugate were plotted as sperm with reacted acrosomes.

### Artificial insemination protocol

During the breeding season, a total of 174 adult ewes were used and randomly distributed in the five experimental groups described above (ASP15: *n* = 38, ASP: *n* = 34, Ø: *n* = 32, ASP Supp.: *n* = 36, and Supp.: *n* = 34). Females were subjected to estrus synchronization using intravaginal sponges with 20 mg fluorogestone acetate (Chronogest^®^, MSD Animal Health, Salamanca, Spain) over 14 days. At sponge withdrawal, ewes received an intramuscular injection of 500 IU eCG (Folligon^®^, MSD Animal Health, Salamanca, Spain). Cervical artificial insemination was performed by experienced technicians at 54 ± 2 h from sponge removal. Animals were placed with the hindquarter upwards, and the semen (400 × 10^6^ sperm) was deposited in the entrance of the cervix using a vaginoscope with an integrated light source and an ovine artificial insemination catheter (IMV Tecnhologies, L’Aigle, France). Reproductive success was evaluated in terms of fertility according to the pregnant ewes 33–37 days post-artificial insemination using an ultrasound scanner (SonoSite 180 Plus Portable Ultrasound, Bothell, WA, USA) equipped with a 7 − 4 MHz convex-array transducer.

### Statistical analysis

All statistical analyses were performed using the SAS/STAT^®^ V 9.1 statistical package (SAS Institute, Cary, NC, USA). Graphs were obtained using Prism 9 (GraphPad Software, San Diego, CA, USA). The normality of variables was examined, and normally distributed data were analyzed using a mixed linear model (MIXED procedure). The same males were analyzed in each experimental group. Fertility data were analyzed as binomial using the GENMOD procedure considering the male as a random factor. Results are displayed as mean ± SEM (Standard Error of the Mean). Differences were considered to be statistically significant at *P* < 0.05.

## Results

### Experiment 1: Seminal plasma addition to epididymal sperm

#### Sperm motility and kinetic parameters

**Fig. 3:**
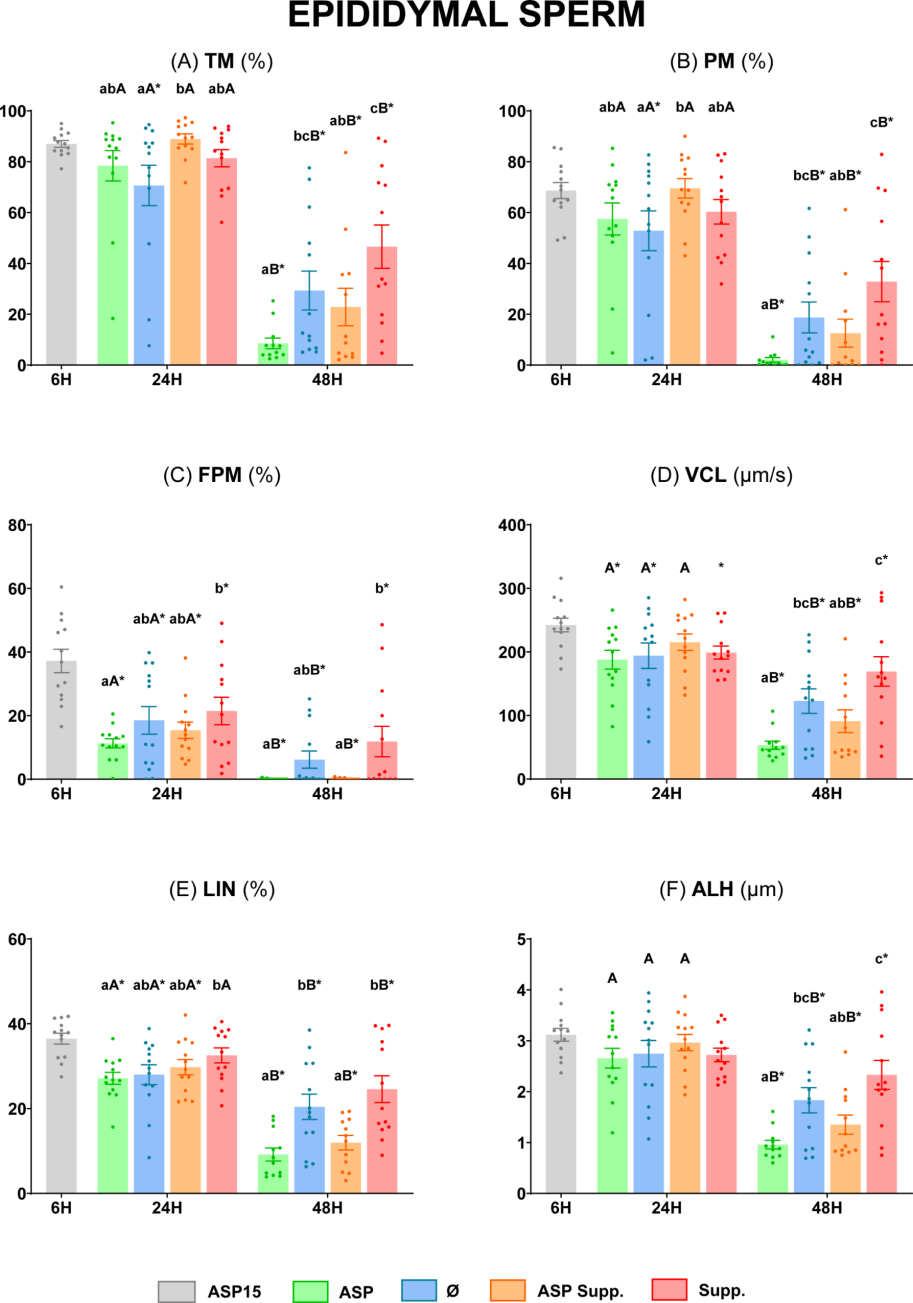
Motility and kinetic parameters of ram epididymal sperm diluted in INRA 96^®^. **(A)** Total motility (TM, %); **(B)** Progressive motility (PM, %); **(C)** Fast progressive motility (FPM, %); **(D)** Curvilinear velocity (VCL, µm/s); **(E)** Linearity (LIN, %); **(F)** Amplitude of lateral head displacement (ALH, µm). The same 13 males were analyzed in each experimental group: epididymal sperm with seminal plasma at 15 °C (**ASP15**), epididymal sperm with seminal plasma at 5 °C (**ASP**), epididymal sperm at 5 °C (**Ø**), epididymal sperm with seminal plasma at 5 °C supplemented (**ASP Supp.**), and epididymal sperm at 5 °C supplemented (**Suppl.**). Graph dots represent the individual values of each ram. Different lowercase letters (a, b, c) indicate significant differences (*P* < 0.05) among the different experimental groups at each evaluation time. Different capital letters (A, B) indicate significant differences (*P* < 0.05) between the 24 and 48 h of evaluation in each experimental group. Asterisk (*) indicates significant differences (*P* < 0.05) between the different experimental groups and ASP15

All the sperm motility and kinetic parameters were similar between the ASP and Ø groups (*P* ≥ 0.05) at 24 h (Fig. 3). In contrast, after 48 h of storage at 5 °C, TM, PM, VCL, LIN, and ALH were significantly higher (*P* < 0.05) in the Ø group compared to the ASP group (Fig. 3, **Panels A**,** B**,** D**,** E**,** and F**), with non-significant differences (*P* ≥ 0.05) in terms of FPM (Fig. 3, **Panel C**). Moreover, the group Supp. showed the best results at this time for the above parameters (*P* < 0.05), while the ASP Supp. group presented values comparable to those of ASP (*P* ≥ 0.05) (Fig. 3, **Panels A**,** B**,** D**,** E**,** and F**).

#### Sperm functionality

**Fig. 4:**
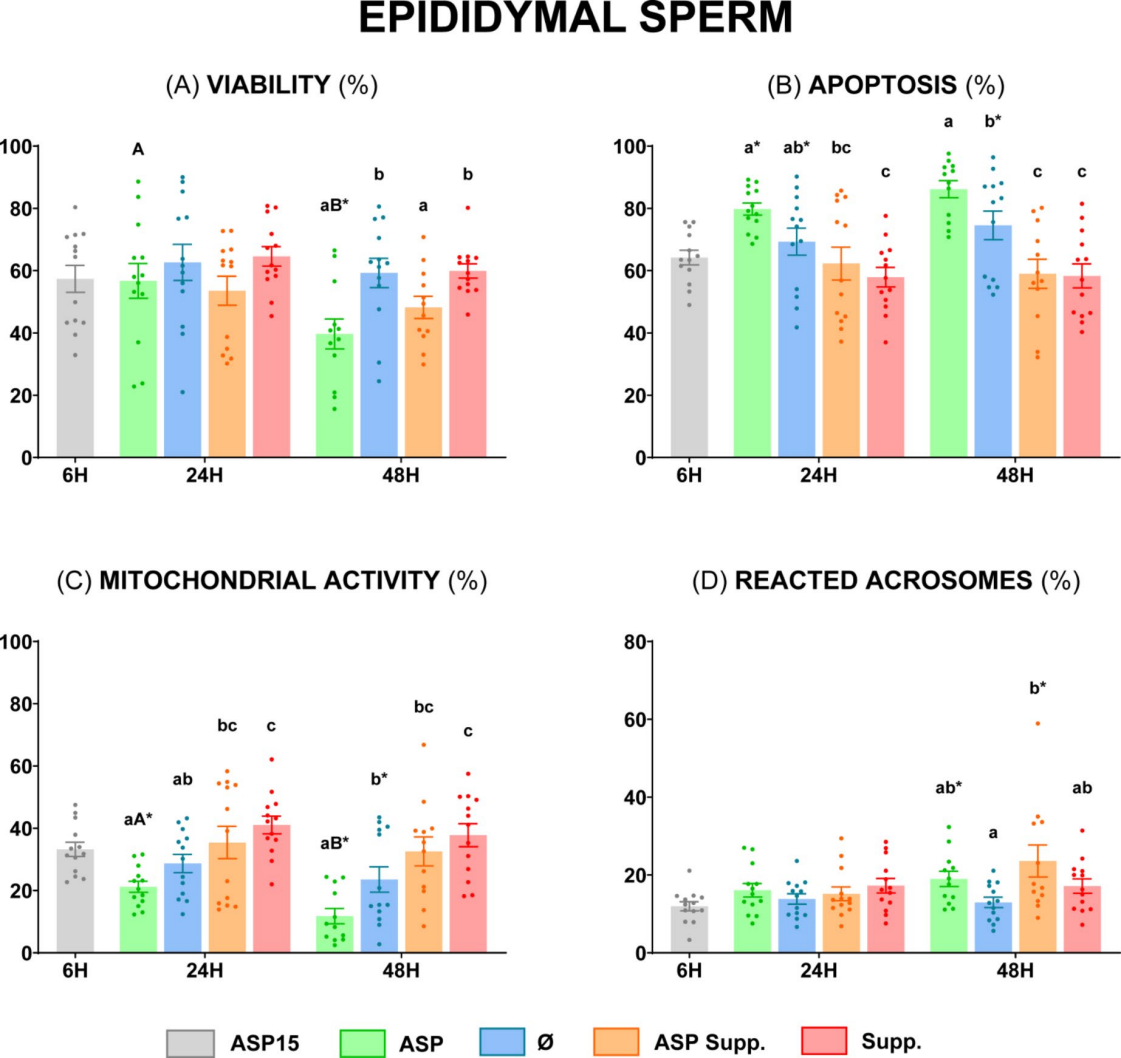
Sperm functionality of ram epididymal sperm diluted in INRA 96^®^. **(A)** Viable sperm (%) (Zombie Violet™); **(B)** Apoptotic sperm (%) (CellEvent™ Caspase-3/7 Green); **(C)** Sperm with high mitochondrial activity (%) (CellROX™ Deep Red); **(D)** Sperm with reacted acrosomes (%) (PNA Alexa Fluor™ 488 Conjugate). The same 13 males were analyzed in each experimental group: epididymal sperm with seminal plasma at 15 °C (**ASP15**), epididymal sperm with seminal plasma at 5 °C (**ASP**), epididymal sperm at 5 °C (**Ø**), epididymal sperm with seminal plasma at 5 °C supplemented (**ASP Supp.**), and epididymal sperm at 5 °C supplemented (**Suppl.**). Graph dots represent the individual values of each ram. Different lowercase letters (a, b, c) indicate significant differences (*P* < 0.05) among the different experimental groups at each evaluation time. Different capital letters (A, B) indicate significant differences (*P* < 0.05) between the 24 and 48 h of evaluation in each experimental group. Asterisk (*) indicates significant differences (*P* < 0.05) between the different experimental groups and ASP15

There were non-significant (*P* ≥ 0.05) differences for any of the sperm functionality parameters evaluated between groups ASP and Ø in the samples stored for 24 h at 5 °C (Fig. 4). However, group Ø showed significantly higher (*P* < 0.05) sperm viability and mitochondrial activity (Fig. 4, **Panels A and C**) and significantly lower (*P* < 0.05) apoptosis (Fig. 4, **Panel B**) in comparison with the ASP group at 48 h. Moreover, sperm viability and reacted acrosomes of Ø after 48 h were similar (*P* ≥ 0.05) to those of ASP15 (Fig. 4, **Panels A and D**). On the other hand, both groups supplemented with SP 30 min before sperm quality evaluation (ASP Supp. and Supp.) revealed the lowest apoptosis and the highest mitochondrial activity values after 24 and 48 h (*P* < 0.05) (Fig. 4, **Panels B and C**), without significant differences (*P* ≥ 0.05) with the ASP15 group.

### Experiment 2: Seminal plasma removal of ejaculated sperm

#### Sperm motility and kinetic parameters

**Fig. 5:**
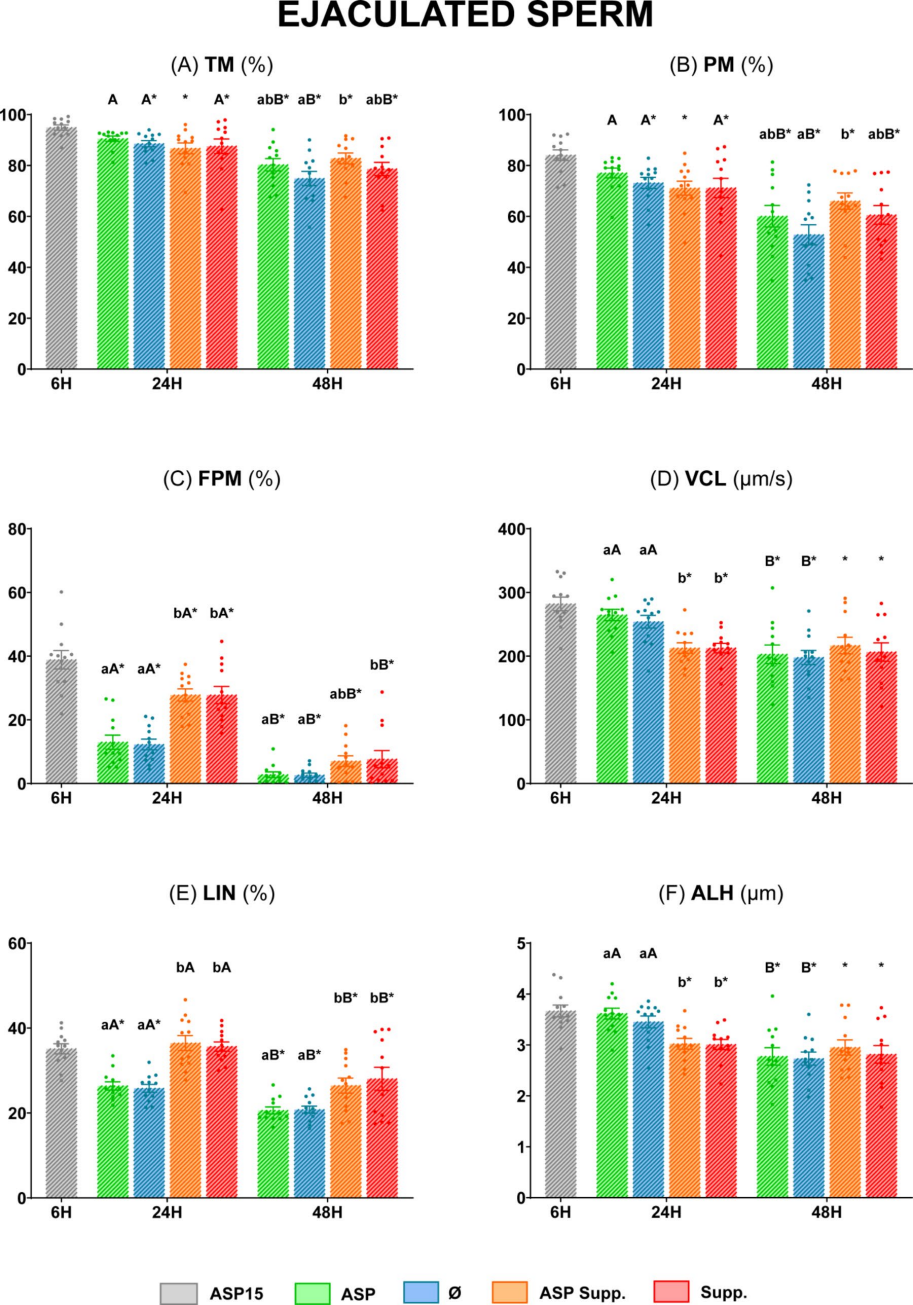
Sperm motility and kinetic parameters of ram ejaculated sperm diluted in INRA 96^®^. **(A)** Total motility (TM, %); **(B)** Progressive motility (PM, %); **(C)** Fast progressive motility (FPM, %); **(D)** Curvilinear velocity (VCL, µm/s); **(E)** Linearity (LIN, %); **(F)** Amplitude of lateral head displacement (ALH, µm). The same 12 males were analyzed in each experimental group: semen at 15 °C (**ASP15**), semen at 5 °C (**ASP**), ejaculated sperm at 5 °C (**Ø**), semen at 5 °C supplemented (**ASP Supp.**), and ejaculated sperm at 5 °C supplemented (**Supp.**). Graph dots represent the individual values of each ram. Different lowercase letters (a, b) indicate significant differences (*P* < 0.05) among the different experimental groups at each evaluation time. Different capital letters (A, B) indicate significant differences (*P* < 0.05) between the 24 and 48 h of evaluation in each experimental group. Asterisk (*) indicates significant differences (*P* < 0.05) between the different experimental groups and ASP15

Non-significant differences were found between ASP and Ø groups at 24 or 48 h for any of the studied parameters (*P* ≥ 0.05) (Fig. 5). Nevertheless, after 24 h at 5 °C, the ASP Supp. and Supp. showed the significantly highest FPM and LIN (*P* < 0.05) (Fig. 5, **Panels C and E**) and significantly lowest VCL and ALH (*P* < 0.05) (Fig. 5, **Panels D and F**). At 48 h, both supplemented groups maintained the significantly highest LIN values (*P* < 0.05) (Fig. 5, **Panel C**), but only the Supp. group showed a significantly higher percentage of fast progressive sperm concerning ASP (*P* < 0.05) (Fig. 5, **Panel E**).

#### Sperm functionality

**Fig. 6:**
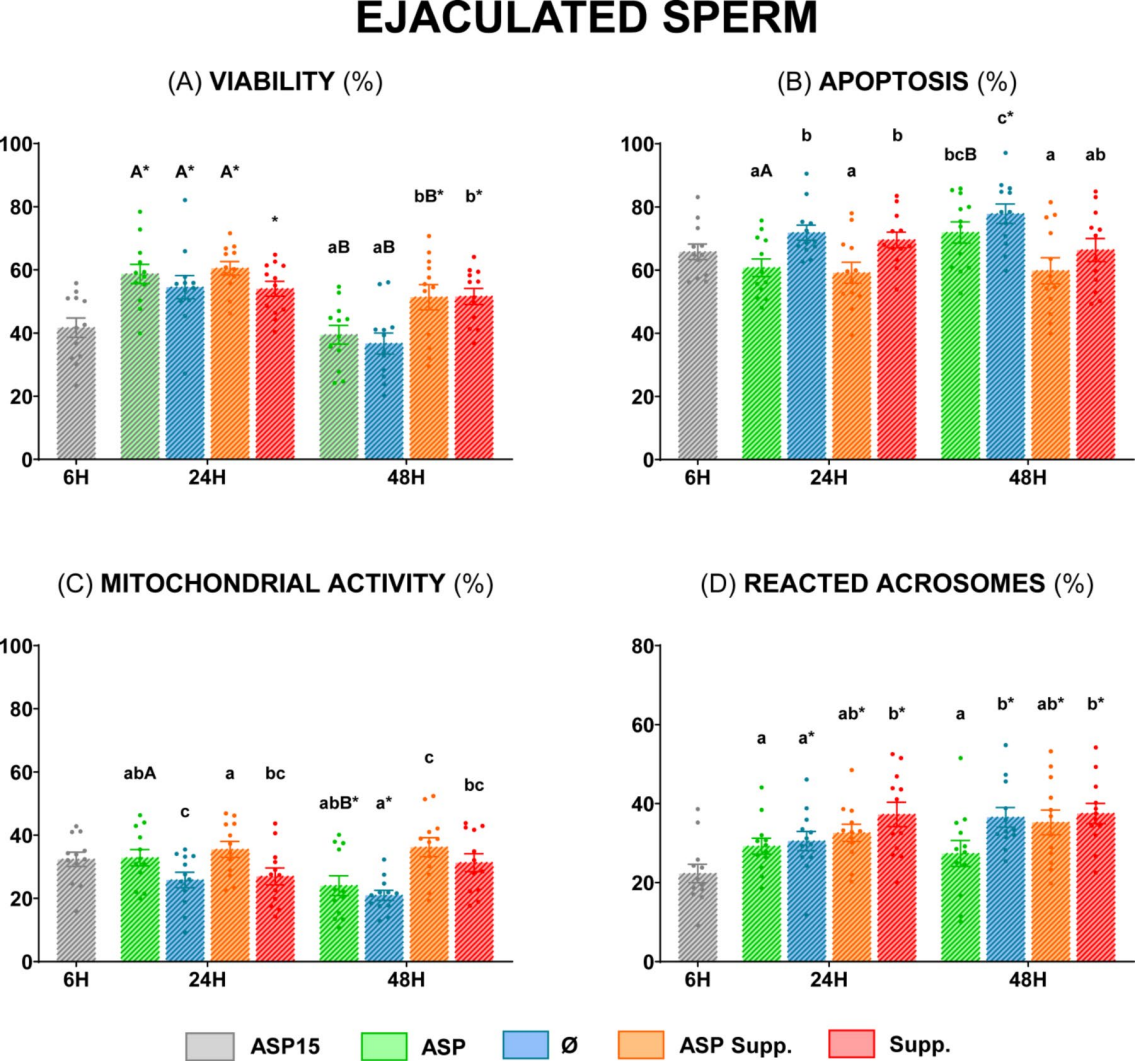
Sperm functionality of ram ejaculated sperm diluted in INRA 96^®^. **(A)** Viable sperm (%) (Zombie Violet™); **(B)** Apoptotic sperm (%) (CellEvent™ Caspase-3/7 Green); **(C)** Sperm with high mitochondrial activity (%) (CellROX™ Deep Red); **(D)** Sperm with reacted acrosomes (%) (PNA Alexa Fluor™ 488 Conjugate). The same 12 males were analyzed in each experimental group: semen at 15 °C (**ASP15**), semen at 5 °C (**ASP**), ejaculated sperm at 5 °C (**Ø**), semen at 5 °C supplemented (**ASP Supp.**), and ejaculated sperm at 5 °C supplemented (**Supp.**). Graph dots represent the individual values of each ram. Different lowercase letters (a, b, c) indicate significant differences (*P* < 0.05) among the different experimental groups at each evaluation time. Different capital letters (A, B) indicate significant differences (*P* < 0.05) between the 24 and 48 h of evaluation in each experimental group. Asterisk (*) indicates significant differences (*P* < 0.05) between the different experimental groups and ASP15.

Samples stored at 5 °C for 24 h revealed significantly higher apoptosis and lower mitochondrial activity in the Ø group than in the ASP group (*P* < 0.05) (Fig. 6, **Panels B and C**), with non-significant differences (*P* ≥ 0.05) in terms of sperm viability or reacted acrosomes (Fig. 6, **Panels A and D**). Regarding the supplemented groups, Supp. showed a significantly higher percentage of reactive acrosomes than ASP and Ø at 24 h (*P* < 0.05) (Fig. 6, **Panel D**). Meanwhile, at 48 h ASP and Ø groups were only significantly different at the level of reacted acrosomes, showing a significantly higher percentage in the Ø group (*P* < 0.05) (Fig. 6, **Panel D**). Furthermore, the ASP Supp. group showed the significantly highest viability and mitochondrial activity (*P* < 0.05) (Fig. 6, **Panels A and C**) and significantly lowest apoptosis at this time (*P* < 0.05) (Fig. 6, **Panel B**), without significant differences (*P* ≥ 0.05) with ASP15 for apoptosis and mitochondrial activity (Fig. 6, **Panels B and C**).

### Experiment 3: Fertility trials

The results of fertility trials are shown in Table 1. At 24 h, we found that fertility significantly increased (*P* < 0.05) in the ASP Supp. group compared to the ASP group. In addition, when comparing all 24-hour experimental groups with ASP15, we found fertility rates significantly lower (*P* < 0.05) in all of them except for the ASP Supp. group (*P* ≥ 0.05).

**Table 1:** Fertility (pregnant ewes/inseminated ewes, %) in the five experimental groups

Experimental group		Fertility	
		%	Pregnant ewes/Total
**6H**	**ASP15**	50.0	19/38
**24H**	**ASP**	17.7^a,^^*^	6/34
	**Ø**	21.9^ab,^^*^	7/32
	**ASP Supp.**	36.1^b^	13/36
	**Supp.**	23.5^ab,^^*^	8/34

**ASP15**: semen with INRA 96^®^ at 15 °C. **ASP**: semen with INRA 96^®^ at 5 °C. **Ø**: ejaculated sperm with INRA 96^®^ at 5 °C. **ASP Supp.**: semen with INRA 96^®^ at 5 °C supplemented. **Supp.**: ejaculated sperm with INRA 96^®^ at 5 °C supplemented. Different lowercase letters (a, b) indicate significant differences (*P* < 0.05) among the experimental groups at 24 h. Asterisk (*) indicates significant differences (*P* < 0.05) between the different experimental groups and ASP15.

## Discussion

Long-term exposure of sperm to the SP during liquid storage could be detrimental to sperm integrity and function in most farm animals [42]. Because of the limited and conflicting information on SP withdrawal for ram sperm refrigeration, this study aimed to investigate if washing ram sperm from SP could be an effective tool to extend sperm lifespan in medium-term preservation in liquid form to optimize artificial insemination protocols in ovine species.

To this end, in Experiment 1 we evaluated the effect of adding autologous SP to ram epididymal sperm in a medium-term preservation protocol (up to 48 h at 5 °C). Sperm remain immotile, metabolically inactive and in a quiescent state in the tail of the epididymis due to a low pH, high CO_2_ tension, low Na^+^/K^+^ rate, and the presence of specific inhibitors of sperm motility [55]. At ejaculation, the mixture of epididymal sperm with SP activates their metabolic activity and motility as a result of the dilution of these inhibitory factors and the provision of activating substances such as inorganic ions, citric acid, organic salts, proteins, and sugars for anaerobic and aerobic metabolism [56]. The expected results were that the ASP group would have higher sperm motility and functionality than the group Ø. Nevertheless, group Ø showed better results than group ASP in most sperm quality parameters after 48 h at 5 °C, without differences between both groups at 24 h. These results agree with all the literature on SP supplementation of ram epididymal sperm during liquid storage regardless of temperature. Rickard and her colleagues [57] established that the presence or absence of SP did not affect the motility of ram epididymal sperm immediately after collection or within a short time (6 h at 37 °C), as we have observed. On the other hand, Dott *et al.* [58] showed that supplementation of ram epididymal sperm with SP had first a stimulatory and then a detrimental effect on motility (following incubation for 22 h at 30 °C). Finally, Rajabi-Toustani *et al.* [48] found a higher percentage of motility and functional membrane integrity in ram epididymal sperm in the absence of SP at 36 h of storage at 5 °C.

These results highlight the potential adverse effect of prolonged exposure to SP on in vitro sperm function. However, since SP has a modulatory role in sperm capacitation and acrosome reaction, sperm-oocyte interaction [59], and female immune response to tolerate sperm and the conceptus [60, 61], we believed it necessary to simulate what happens in natural mating by a final SP supplementation during a brief time (30 min), so we introduce the study groups ASP Supp. and Supp. In this sense, we noted that Supp. group had the highest sperm motility and kinetic parameters at 48 h, as well as greater viability and mitochondrial activity than the other experimental groups. This may probably be explained because SP proteins have been found to reverse cold-shock damage on ram sperm membrane [62], even improving characteristics of frozen-thawed ram sperm such as motility, capacitation and ability to penetrate cervical mucus in vitro [63, 64].

From all the above, we could conclude that SP privation indeed would be beneficial during a medium-term ram sperm preservation protocol in liquid form, as well as a supplementation in a final moment. Therefore, we conducted Experiment 2 to evaluate the effect of SP removal on ram semen under the same storage conditions as in Experiment 1 using the centrifugation protocol previously designed by our research group: 1,200 × *g* for 10 min at 15 °C [49]. Surprisingly, no changes in motility were observed when SP was removed at any evaluation time. Instead, SP withdrawal appeared harmful to ram sperm functionality, expressing increased apoptosis and decreased mitochondrial activity after 24 h at 5 °C and a higher percentage of reacted acrosomes at 48 h.

The negative effect on the quality of washed ram sperm was also observed by Mata-Campuzano and her collaborators [46], but in a different way. They obtained a lower percentage of progressive motility at 3 and 24 h of storage in the 0% SP group, without differences in sperm viability. By contrast, the results from the current study and that of Mata-Campuzano and her colleagues [46] differ from Paul *et al.* [47] and Rajabi-Toustani *et al.* [48], who showed that most of the sperm motility and kinetic attributes, as well as the viability, membrane integrity and non-capacitated sperm count, were improved in the SP removal groups. This fact could be explained by considering the different methods employed for SP elimination. It is possible that our SP removal protocol could be less effective than the protocols by high dilution (1:15 and 200 × 10^6^ sperm/mL), followed or not by a centrifugal washing at 150 × *g* for 10 min at room temperature [47], or only by centrifugation of ejaculates at 720 × *g* for 10 min at room temperature [48]. Nevertheless, none of the methods used in the above works for SP withdrawal could be applied in field conditions. Because of the particular anatomy of the ovine cervical canal, the sperm dose for cervical artificial insemination should have a limited volume (< 0.25 mL) with a relatively large number of sperm (400 × 10^6^ sperm) to avoid a possible backflow [65]. Removal of SP only by high dilution would not represent a feasible option in artificial insemination procedures due to the high volume and low sperm concentration involved. In turn, the centrifugation process using low centrifugal forces at room temperature, preceded or not by high dilutions, would be ruled out since our research group has demonstrated a large sperm loss under these conditions, decreasing technique yield [66].

Regarding the final supplementation with SP, we observed again its positive effect. Both supplemented groups (ASP Supp. and Supp.) displayed better LIN, viability, apoptosis, and mitochondrial activity at 48 h. At 24 h, on the other hand, group ASP Supp. had the highest FPM, LIN, and mitochondrial functionality and the lowest apoptosis.

The results of the fertility trials partly coincided with those of the in vitro analysis of sperm quality performed in Experiment 2. SP supplementation of the ejaculate processed as usual improves pregnancy rates in females cervically inseminated with ram semen stored at 5 °C for 24 h, and more importantly, reaching similar levels to the 6 h (50.0% ASP15, 17.7% ASP, and 36.1% ASP Supp.). The increased fertility after a 24-hour sperm preservation protocol by adding 30% ram SP had been previously noted by López-Pérez and Pérez-Clariget [67] in ewes cervically inseminated using a Tris-egg yolk-based extender, with the difference that SP inclusion was performed at semen dilution in the beginning of the sperm preservation protocol. On the other hand, Maxwell and his colleagues [68] showed similar percentages of pregnant ewes after cervical artificial insemination with fresh sperm in the presence or absence of 30% SP in the medium. Also, Belibasaki *et al.* [69] registered no changes in the percentage of lambed ewes, but there was an increase in litter size using ram semen diluted with 50% skim milk and 50% SP (6 h at 16 °C) for intracervical artificial insemination at the peak of the breeding season, indicating that SP supplementation can influence the fertility of ewes or the fertilizing capacity of extended ram semen.

## Conclusion

To summarize, SP absence was beneficial for a medium-term preservation protocol (up to 48 h at 5 °C) of ram epididymal sperm, but the same preservation protocol for ram ejaculated sperm revealed a possible failure of the SP removal method in avoiding the sperm-SP interaction effect. Meanwhile, SP supplementation of ram semen at the end of the preservation protocol increased in vitro sperm quality and fertility after artificial insemination. These findings highlight the modulating role of SP on ram sperm quality and fertilization ability and pave the way for improving medium-term semen preservation in the ovine species.

## Data Availability

The datasets obtained and/or analyzed during the current study are available from the corresponding author upon reasonable request.
